# Endoscopic Ultrasound‐Guided Hepaticogastrostomy With Plastic Stents in Comparison to Transpapillary Drainage With Metallic Stents for Unresectable Malignant Distal Biliary Obstructions

**DOI:** 10.1002/deo2.70263

**Published:** 2026-01-11

**Authors:** Hidehito Sumiya, Yoshihide Kanno, Shinsuke Koshita, Takahisa Ogawa, Hiroaki Kusunose, Toshitaka Sakai, Keisuke Yonamine, Kazuaki Miyamoto, Fumisato Kozakai, Haruka Okano, Kento Hosokawa, Shun Nozaki, Kei Ito

**Affiliations:** ^1^ Department of Gastroenterology Sendai City Medical Center Miyagi Japan

**Keywords:** antegrade stenting, endoscopic retrograde cholangiopancreatography, endoscopic ultrasound‐guided hepaticogastrostomy, malignant distal biliary obstruction, plastic stent

## Abstract

**Objectives:**

Endoscopic ultrasound‐guided hepaticogastrostomy (EUS‐HGS) is usually performed for unresectable malignant distal biliary obstruction (MDBO) when endoscopic retrograde cholangiopancreatography‐guided biliary stenting with fully covered self‐expandable metallic stents (EBS‐MSs) fails. We aimed to clarify the clinical outcomes of EUS‐HGS with plastic stents (HGS‐PSs) compared to EBS‐MS.

**Methods:**

We retrospectively reviewed patients who underwent either HGS‐PS with or without antegrade stenting using MS (AS‐MS) or EBS‐MS as initial biliary drainage for unresectable MDBO between January 2017 and July 2024.

**Results:**

A total of 27 patients were included in the HGS‐PS group, and 128 patients were included in the EBS‐MS group. Median procedure time was significantly shorter for the HGS‐PS group (24 vs. 39 min, *p* < 0.001), and the incidence of adverse events was comparable (22% vs. 32%, *p* = 0.365). The HGS‐PS group had a significantly higher recurrent biliary obstruction (RBO) rate (48% vs. 26%, *p* = 0.002) and shorter time to RBO (TRBO) (169 vs. 341 days, *p* = 0.001). After propensity score matching, no significant differences were observed in either the RBO rate or TRBO. Subgroup analyses showed that TRBO was comparable between the HGS‐PS with AS‐MS and EBS‐MS groups (273 vs. 341 days, *p* = 0.609).

**Conclusions:**

Although TRBO tended to be shorter for HGS‐PS compared to EBS‐MS, the addition of AS‐MS to HGS‐PS led to comparable TRBO, suggesting that this combination may be a viable alternative.

Clinical Trial Registration: The authors have confirmed clinical trial registration is not needed for this submission.

## 1 Introduction

Endoscopic retrograde cholangiopancreatography‐guided biliary stenting (EBS) is the standard initial treatment for malignant distal biliary obstructions (MDBO). Since fully covered self‐expandable metallic stents (FCSEMSs) provide a longer time to recurrent biliary obstruction (TRBO) than plastic stents (PSs), FCSEMSs (EBS‐MSs) are recommended for unresectable MDBO [1, 2]. However, EBS can fail due to an endoscopically inaccessible duodenal papilla caused by tumor invasion or difficulty in biliary cannulation. In such cases, endoscopic ultrasound (EUS)‐guided biliary drainage (EUS‐BD), including EUS‐guided hepaticogastrostomy (EUS‐HGS), has been performed as a salvage option [3, 4, 5].

In EUS‐HGS, both PSs and SEMSs are usable for placement at the endosonographically created route (ESCR) [6], each with distinct advantages and limitations. EUS‐HGS with PSs (HGS‐PSs) offers several practical advantages. They are relatively easy to deploy, available in various shapes, and allow selection based on anatomical characteristics. Since PSs do not completely obstruct side branches, they are associated with lower risks of segmental cholangitis, liver abscess, and cholecystitis than MSs. Another key benefit is easier stent removal when occluded. In contrast, MSs, particularly laser‐cut types, are extremely difficult to remove. Braided, partially covered MSs may become embedded due to tissue ingrowth at the uncovered segment, making removal nearly impossible. Even with fully covered MSs, reintervention can be challenging if tissue hyperplasia develops at the hepatic end of the stent. Although PSs may result in shorter TRBO than EUS‐HGS with SEMSs (HGS‐MSs) [7, 8, 9], the addition of EUS‐guided antegrade stenting (EUS‐AS) can compensate for this limitation [10]. Thus, PSs are often preferred when ease and flexibility of reintervention are prioritized, and their use remains common in some regions [8, 11, 12, 13].

Recently, EUS‐BD has been reported as a promising alternative to EBS‐MS for initial biliary drainage of MDBO due to its favorable efficacy and safety [14, 15, 16, 17, 18]. However, these previous reports have focused mainly on EUS‐guided choledochoduodenostomy (EUS‐CDS), and comparative studies between EUS‐HGS and EBS‐MS remain scarce, particularly for HGS‐MS [19, 20, 21]. Given the increasing interest in the potential utility of HGS‐PS, it is important to evaluate its clinical significance compared with EBS‐MS. Therefore, we clarified the clinical outcomes of HGS‐PS, including EUS‐AS with SEMSs (AS‐MS), and EBS‐MS as initial biliary drainage for unresectable MDBO.

## 2 Methods

### 2.1 Study Design and Ethics Statement

This single‐center retrospective study was conducted at Sendai City Medical Center and approved by its institutional review board (approval number: 2024–0064). Written informed consent for the procedures was obtained from all patients, and participation in this study was approved through an opt‐out process on the hospital website.

### 2.2 Patients

**FIGURE 1:**
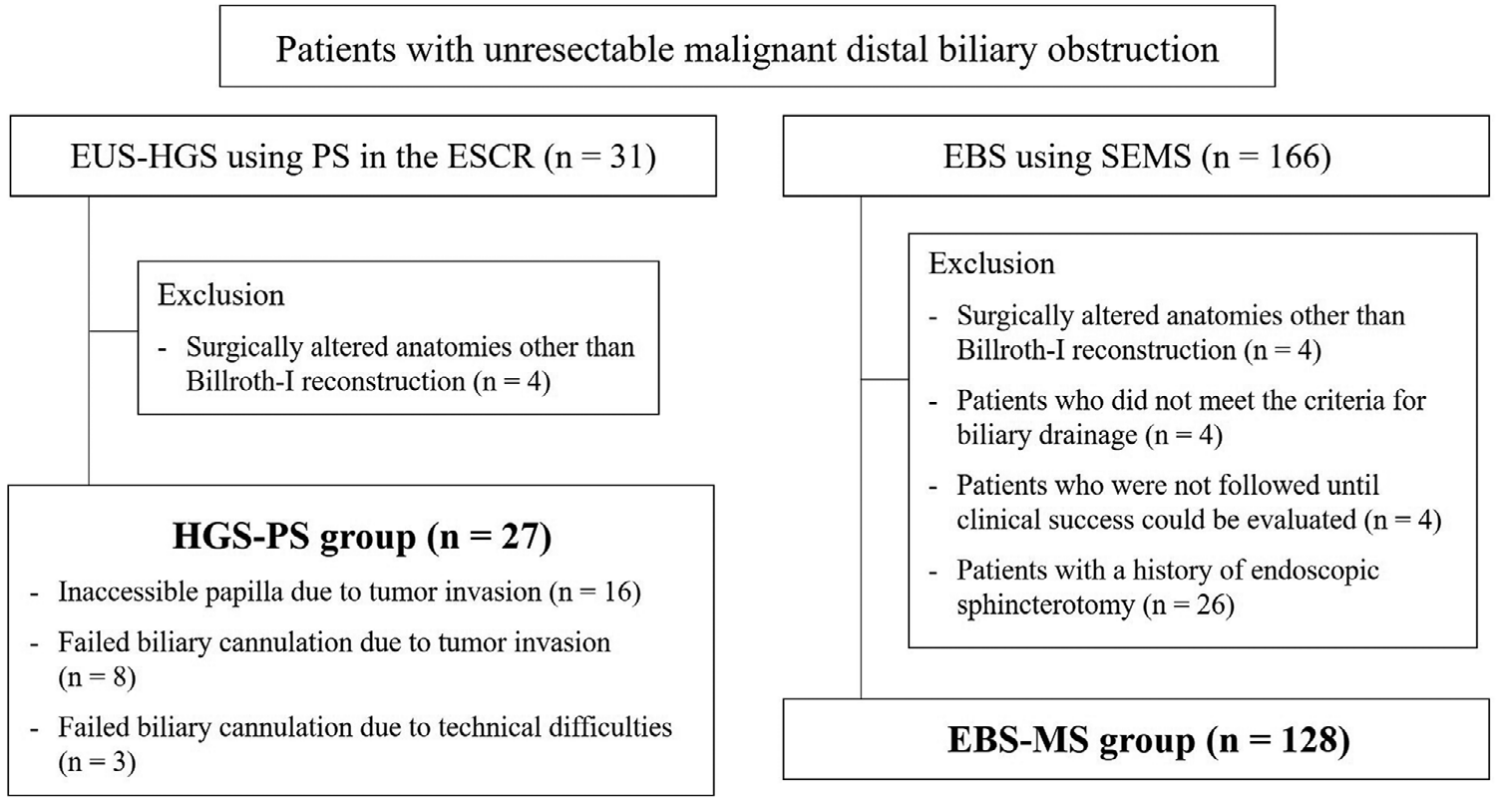
Flowchart outlining the study protocol. EUS‐HGS, endoscopic ultrasound‐guided hepaticogastrostomy; PS, plastic stent; ESCR, endosonographically/EUS‐guided created route; EBS, endoscopic retrograde cholangiopancreatography‐guided biliary stenting; SEMS, self‐expandable metallic stent.

From an endoscopic prospectively registered database in our hospital, patients with unresectable MDBO who underwent either HGS‐PS or EBS‐MS as initial biliary drainage between January 2017 and July 2024 were included in this study. EBS was generally attempted or performed in all cases. When EBS failed due to an inaccessible papilla or unsuccessful biliary cannulation caused by tumor invasion or technical difficulties, subsequent EUS‐HGS was performed. Patients who met the following criteria were excluded from the analysis: 1) patients with surgically altered anatomies other than Billroth‐I reconstruction, 2) those without obstructive jaundice (total bilirubin ≥ 2.0 mg/dL) or elevated levels of liver enzymes (aspartate aminotransferase or alanine aminotransferase ≥ 100 IU/L), 3) those who were not monitored until clinical success could be evaluated, and 4) those with a history of endoscopic sphincterotomy (EST). HGS‐PS combined with AS‐MS was included. Finally, 27 patients in the HGS‐PS group and 128 in the EBS‐MS group were included in the analysis (Figure 1).

### 2.3 Endoscopic Procedures

Rectal nonsteroidal anti‐inflammatory drugs (diclofenac sodium, 25 or 50 mg/body) were routinely administered to prevent post‐ERCP pancreatitis in the EBS‐MS group and in the HGS‐PS group when AS‐MS was intended.

**FIGURE 2:**
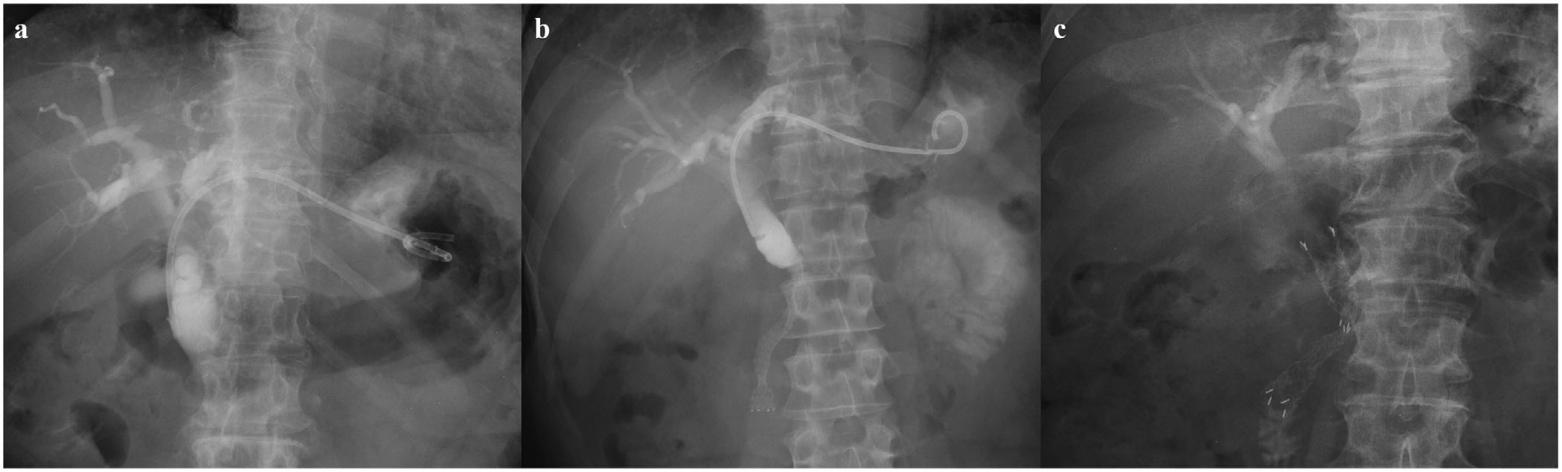
Fluoroscopic X‐ray imaging of endoscopic biliary stenting for malignant distal biliary obstructions. (a) HGS‐PS. (b) HGS‐PS with AS‐MS. (c) EBS‐MS. HGS‐PS, endoscopic ultrasound‐guided hepaticogastrostomy with a plastic stent; AS‐MS, endoscopic ultrasound‐guided antegrade stenting with a self‐expandable metallic stent; EBS‐MS, endoscopic retrograde cholangiopancreatography‐guided biliary stenting with a self‐expandable metallic stent.

In the HGS‐PS group, either the B2 or B3 intrahepatic bile duct was punctured using a 19‐ or 22‐gauge needle, EZ Shot 3 Plus (Olympus Co., Tokyo, Japan) or Expect (Boston Scientific Japan K.K., Tokyo, Japan), followed by insertion of a guidewire into the bile duct. After tract dilation using a bougie, balloon, or drill dilator, the stricture of the bile duct was evaluated using cholangiography. One of the following 7‐Fr PSs was placed in the ESCR: Through & Pass, Type IT (Gadelius Medical K.K., Tokyo, Japan) and Flexima, straight type (Boston Scientific Japan K.K.) (Figure 2a). In almost all recent cases, Type IT stents with a proximal pigtail to prevent migration were used. When simultaneous AS‐MS was attempted at the operator's discretion, a guidewire was advanced beyond the stricture. For AS‐MS, one of the following uncovered SEMS (UCSEMS) or FCSEMSs was placed across or above the papilla before placing the PS in the ESCR: ZEO STENT V (ZEON MEDICAL INC., Tokyo, Japan); YABUSAME (Kaneka Medix, Osaka, Japan); Zilver635 (Cook Medical Japan, Tokyo, Japan); BileRush (PIOLAX MEDICAL DEVICES, INC., Kanagawa, Japan); HANAROSTENT (Boston Scientific Japan K.K.); and X‐suit NIR (Olympus Co.) (Figure 2b).

In the EBS‐MS group, EST was performed before EBS when not contraindicated. Biopsies or intraductal ultrasonography (IDUS) were performed when necessary. An FCSEMS was placed across or above the papilla. Simultaneous endoscopic transpapillary gallbladder drainage (ETGBD) was permitted using an IYO‐stent (Gadelius Medical K.K.) to prevent cholecystitis. The diameters of the FCSEMSs ranged from 6 to 12 mm. One of the following FCSEMSs was placed: HANAROSTENT; Niti‐S (Century Medical, Inc., Tokyo, Japan); BONASTENT (Medico's Hirata Inc., Tokyo, Japan); BONASTENT M‐Intraductal (Medico's Hirata Inc.); Evolution (Cook Medical Japan); HILZO (ZEON MEDICAL INC.); and X‐suit NIR (Figure 2c).

The selection of biliary stents and the decision to add AS‐MS or ETGBD were conducted at the operator's discretion. All procedures were performed as the initial biliary drainage in a single session. No scheduled stent exchanges were performed.

### 2.4 Outcome Measures and Definitions

The primary outcome was TRBO. Secondary outcomes included procedure time, clinical success, RBO, non‐RBO adverse events (AEs), and overall survival (OS). These outcomes were retrospectively compared between the HGS‐PS and EBS‐MS groups. To ensure comparability between cohorts, propensity score matching (PSM) was performed. In addition, subgroup analyses were performed as follows: 1) comparison of the HGS‐PS with and without AS‐MS groups, and 2) comparison of the HGS‐PS with the AS‐MS and EBS‐MS groups.

The diagnosis of concomitant cholangitis was made based on the diagnostic criteria of the Tokyo Guidelines 2018 [22]. Duodenal invasion was defined as symptomatic stenosis requiring stent placement before or during the same hospitalization. An operator was defined as a trainee if their cumulative experience included fewer than 30 EUS‐HGS or 200 ERCP procedures [23, 24]. Procedure time was defined as the time from puncture to stent placement in the HGS‐PS group and from biliary cannulation to stent placement in the EBS‐MS group. The time spent on biopsies and IDUS was excluded from the procedure time. Clinical success, RBO, non‐RBO AEs, TRBO, and OS were evaluated based on the TOKYO criteria 2024 [6].

### 2.5 Statistical Analyses

All statistical analyses were performed using EZR [25]. A Pearson chi‐square test or Fisher's exact test was used for the categorical variables, whereas Student's t‐test or Mann‐Whitney U test was used for continuous data. TRBO and OS were analyzed using the Kaplan‐Meier method and a log‐rank test, and the results are presented as the median and 95% confidence intervals. PSM was performed to balance baseline patient characteristics, including age, sex, performance status, primary disease, previous cholecystectomy, administration of antithrombotic agents, concomitant cholangitis, metastasis, chemotherapy, and duodenal invasion. Propensity scores were generated using logistic regression analysis. One‐to‐one matching without replacement was performed with a caliper width of 0.2. A *p*‐value of < 0.05 was considered statistically significant.

## 3 Results

### 3.1 Patient Characteristics

The mean age of the patients was 76 ± 8 years. The primary disease differed significantly between the two groups (*p* = 0.006), with pancreatic cancer more common in the EBS‐MS group. Duodenal invasion was significantly more frequent in the HGS‐PS group (*p* < 0.001), as EUS‐HGS was performed when the duodenoscope could not reach the duodenal papilla. Surgical interventions, such as gastrojejunostomy, were not performed for duodenal invasion. Other baseline characteristics did not differ significantly between groups (Table 1).

**TABLE 1:** Patient characteristics.

	HGS‐PS	EBS‐MS	*p‐*Value
	*n* = 27	*n* = 128	
Age, years, mean ± SD	77 ± 11	76 ± 11	0.823
Sex, male/female	16/11	51/77	0.087
Performance status, median (range)	0 (0–3)	1 (0–4)	0.557
Primary disease, *n* (%)			0.006
Pancreatic cancer	18 (67)	107 (84)	
Biliary cancer	4 (15)	16 (13)	
Gastric cancer	3 (11)	0	
Others	2 (7.4)	5 (3.9)	
Previous cholecystectomy, *n* (%)	3 (11)	4 (3.1)	0.102
Administration of antithrombotic agents, *n* (%)	1 (3.7)	17 (13)	0.202
Concomitant cholangitis, *n* (%)	9 (33)	32 (25)	0.471
Metastasis, *n* (%)	15 (56)	71 (56)	1.000
Chemotherapy, *n* (%)	13 (48)	60 (47)	1.000
Duodenal invasion, *n* (%)	6 (22)	1 (0.8)	< 0.001

EBS‐MS, endoscopic retrograde cholangiopancreatography‐guided biliary stenting with self‐expandable metallic stents; HGS‐PS, endoscopic ultrasound‐guided hepaticogastrostomy with plastic stents; SD, standard deviation.

### 3.2 Endoscopic Procedures

In the HGS‐PS group, a B3 duct was punctured in 85% of patients. AS‐MS was conducted in 44%, for most of whom 10‐mm UCSEMSs were used. In the EBS‐MS group, 10‐mm FCSEMSs were commonly used (91%). Biopsies and IDUS were performed in some cases, whereas mapping biopsy with peroral cholangioscopy was not performed.

Operator experience did not differ significantly between groups. Procedure time was significantly shorter for the HGS‐PS group (24 vs. 39 min, *p* < 0.001) (Table 2).

**TABLE 2:** Endoscopic procedures.

	HGS‐PS	EBS‐MS	*p*‐Value
	*n* = 27	*n* = 128	
Operator, expert/trainee	18/9	67/61	0.205
Procedure time, minutes, median (range)	24 (9–87)	39 (9–101)	< 0.001
**HGS‐PS**			
Punctured biliary tract, B2/B3	4/23		
Diameter of punctured biliary tract, mm, median (range)	6 (2–10)		
Diameter of PS in the ESCR, *n* (%)			
7 Fr	27 (100)		
AS‐MS, *n* (%)	12 (44)		
Type of AS, UCSEMS/FCSEMS	10/2		
Diameter of AS, *n* (%)			
6 mm	1 (8.3)		
8 mm	2 (17)		
10 mm	9 (75)		
The way of placement of AS, across/above the papilla	9/3		
**EBS‐MS**			
Biopsy, *n* (%)		30 (23)	
Intraductal ultrasonography, *n* (%)		4 (3.1)	
Diameter of MS, *n* (%)			
6 mm		1 (0.8)	
8 mm		9 (7)	
10 mm		116 (91)	
12 mm		2 (1.6)	
Placement method for MSs, across/above the papilla		119/9	
ETGBD, *n* (%)		10 (7.8)	

AS‐MS, endoscopic ultrasound‐guided antegrade stenting with self‐expandable metallic stents; EBS‐MS, endoscopic retrograde cholangiopancreatography‐guided biliary stenting with self‐expandable metallic stents; ESCR, endosonographically created route; ETGBD, endoscopic transpapillary gallbladder drainage; FCSEMS, fully‐covered self‐expandable metallic stent; HGS‐PS, endoscopic ultrasound‐guided hepaticogastrostomy with plastic stents; UCSEMS, uncovered self‐expandable metallic stent.

### 3.3 Clinical Outcomes of Endoscopic Biliary Drainage

**FIGURE 3:**
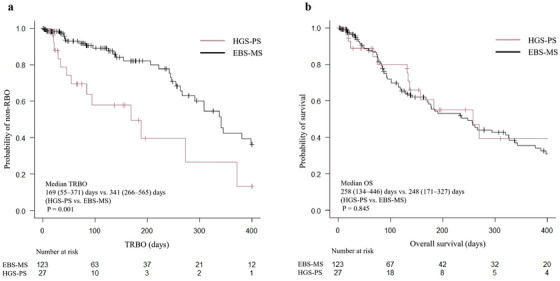
Kaplan‐Meier analysis of the probability of non‐RBO and survival comparing the HGS‐PS and EBS‐MS groups. (a) The median TRBO was 169 and 341 days for the HGS‐PS and EBS‐MS groups, respectively, and significantly longer for the EBS‐MS group (*p* = 0.001). (b) The median OS was 258 and 248 days in the HGS‐PS and EBS‐MS group, respectively, and there was no significant difference (*p* = 0.845). RBO, recurrent biliary obstruction; HGS‐PS, endoscopic ultrasound‐guided hepaticogastrostomy with a plastic stent; EBS‐MS, endoscopic retrograde cholangiopancreatography‐guided biliary stenting with a self‐expandable metallic stent; TRBO, time to recurrent biliary obstruction; OS, overall survival.

The outcomes are shown in Table 3. The clinical success rate was 100% in the HGS‐PS group and 96% in the EBS‐MS group. The RBO rate was significantly higher in the HGS‐PS group than in the EBS‐MS group (48% [13/27] vs. 26% [32/123], *p* = 0.002). In the HGS‐PS group, the cause of RBO was stent occlusion in 10 cases, stent proximal migration in one case, and kinking of the bile duct due to distal and proximal stent dislocation in one case each. In the EBS‐MS group, RBO was caused by stent occlusion in 28 cases, stent proximal and distal migration in two and one cases, respectively, and kinking of the bile duct due to distal stent dislocation in 1 case. From Kaplan‐Meier analysis, TRBO in the HGS‐PS group was significantly shorter than in the EBS‐MS group (169 [55–371] vs. 341 [266–565] days, *p* = 0.001) (Figure 3a).

**TABLE 3:** Clinical outcomes of endoscopic biliary drainage.

	HGS‐PS	EBS‐MS	*p*‐Value
	*n* = 27	*n* = 128	
Clinical success, *n* (%)	27 (100)	123 (96)	0.588
RBO, *n* (%)	13 (48)	32 (26)	0.002
TRBO, days, median (95%CI)	169 (55–371)	341 (266–565)	0.001
Non‐RBO AEs, *n* (%)	6 (22)	41 (32)	0.365
Early non‐RBO AEs (≤ 2 weeks), mild/moderate/severe	5/1/0	12/14/3	1.000
Acute pancreatitis	1/0/0	8/4/3	0.309
Cholecystitis	0/0/0	1/5/0	0.591
Nonocclusion cholangitis	0/0/0	2/0/0	1.000
Bleeding	0/0/0	0/5/0	0.588
Bile leak	0/0/0	1/0/0	1.000
Peritonitis	4/1/0	0/0/0	< 0.001
Late non‐RBO AEs (> 2 weeks), mild/moderate/severe	0/0/0	1/13/0	0.131
Cholecystitis	0/0/0	1/11/0	0.128
Liver abscess	0/0/0	0/2/0	1.000
Death due to AEs, *n* (%)	0	1 (0.8)	1.000
OS, days, median (95%CI)	258 (134–446)	248 (171–327)	0.845

AEs, adverse events; CI, confidence intervals; EBS‐MS, endoscopic retrograde cholangiopancreatography‐guided biliary stenting with self‐expandable metallic stents; HGS‐PS, endoscopic ultrasound‐guided hepaticogastrostomy with plastic stents; OS, overall survival; RBO, recurrent biliary obstruction; TRBO, time to recurrent biliary obstruction.

The incidence of non‐RBO AEs was similar between the two groups (22% vs. 32%, *p* = 0.365). Peritonitis was more frequent in the HGS‐PS group, whereas cholecystitis occurred more often in the EBS‐MS group. One case of peritonitis required additional EUS‐guided drainage, whereas the remaining cases were relieved by conservative treatment. In the EBS‐MS group, severe post‐ERCP pancreatitis developed in three patients, resulting in one death. Cholecystitis and liver abscesses required percutaneous or EUS‐guided gallbladder drainage except in 1 case. Bleeding was controlled endoscopically, and all other AEs improved with conservative treatment.

The median OS was 258 (134–446) days in the HGS‐PS group and 248 (171–327) days in the EBS‐MS group, and there was no significant difference between the two groups (*p* = 0.845) (Figure 3b).

### 3.4 Clinical Outcomes of the Propensity Score‐Matched Cohort

PSM was performed between the HGS‐PS and EBS‐MS groups to adjust for baseline differences in the patient characteristics, and 17 pairs were selected for evaluation. The outcomes are shown in Table 4. After PSM, no significant differences were observed between the groups in baseline factors such as primary disease or duodenal invasion. AS‐MS was performed for 47% of patients.

**TABLE 4:** Patient characteristics and clinical outcomes of the propensity score‐matched cohort.

	HGS‐PS	EBS‐MS	*p‐*Value
	*n* = 17	*n* = 17	
**Patient characteristics**			
Age, years, mean ± SD	74 ± 11	79 ± 13	0.288
Sex, male/female	8/9	6/11	0.728
Performance status, median (range)	0 (0–3)	1 (0–3)	0.470
Primary disease, *n* (%)			0.562
Pancreatic cancer	13 (77)	10 (59)	
Biliary cancer	3 (18)	5 (29)	
Others	1 (5.9)	2 (12)	
Previous cholecystectomy, *n* (%)	0	1 (5.9)	1.000
Administration of antithrombotic agents, *n* (%)	1 (5.9)	2 (12)	1.000
Concomitant cholangitis, *n* (%)	5 (29)	5 (29)	1.000
Metastasis, *n* (%)	11 (65)	9 (53)	0728
Chemotherapy, *n* (%)	11 (65)	9 (53)	1.000
Duodenal invasion, *n* (%)	0	0	N.A.
**Clinical outcomes**			
AS‐MS, *n* (%)	8 (47)		
Operator, expert/trainee	13/4	9/8	0.285
Procedure time, minutes, median (range)	24 (10–40)	45 (24–79)	< 0.001
Clinical success, *n* (%)	17 (100)	16 (94)	1.000
RBO, *n* (%)	8 (47)	3 (19)	0.173
TRBO, days, median (95%CI)	273 (46–N.A.)	341 (263–N.A.)	0.159
Non‐RBO AEs, *n* (%)	3 (18)	4 (24)	1.000
Acute pancreatitis	0	2 (12)	0.485
Cholecystitis	0	2 (12)	0.485
Perforation	0	1 (5.9)	1.000
Peritonitis	3 (18)	0	0.227
OS, days, median (95%CI)	258 (132–424)	168 (39–N.A.)	0.431

AEs, adverse events; AS‐MS, endoscopic ultrasound‐guided antegrade stenting with self‐expandable metallic stents; CI, confidence intervals; EBS‐MS, endoscopic retrograde cholangiopancreatography‐guided biliary stenting with self‐expandable metallic stents; HGS‐PS, endoscopic ultrasound‐guided hepaticogastrostomy with plastic stents; N.A., not available; OS, overall survival; RBO, recurrent biliary obstruction; SD, standard deviation; TRBO, time to recurrent biliary obstruction.

**FIGURE 4:**
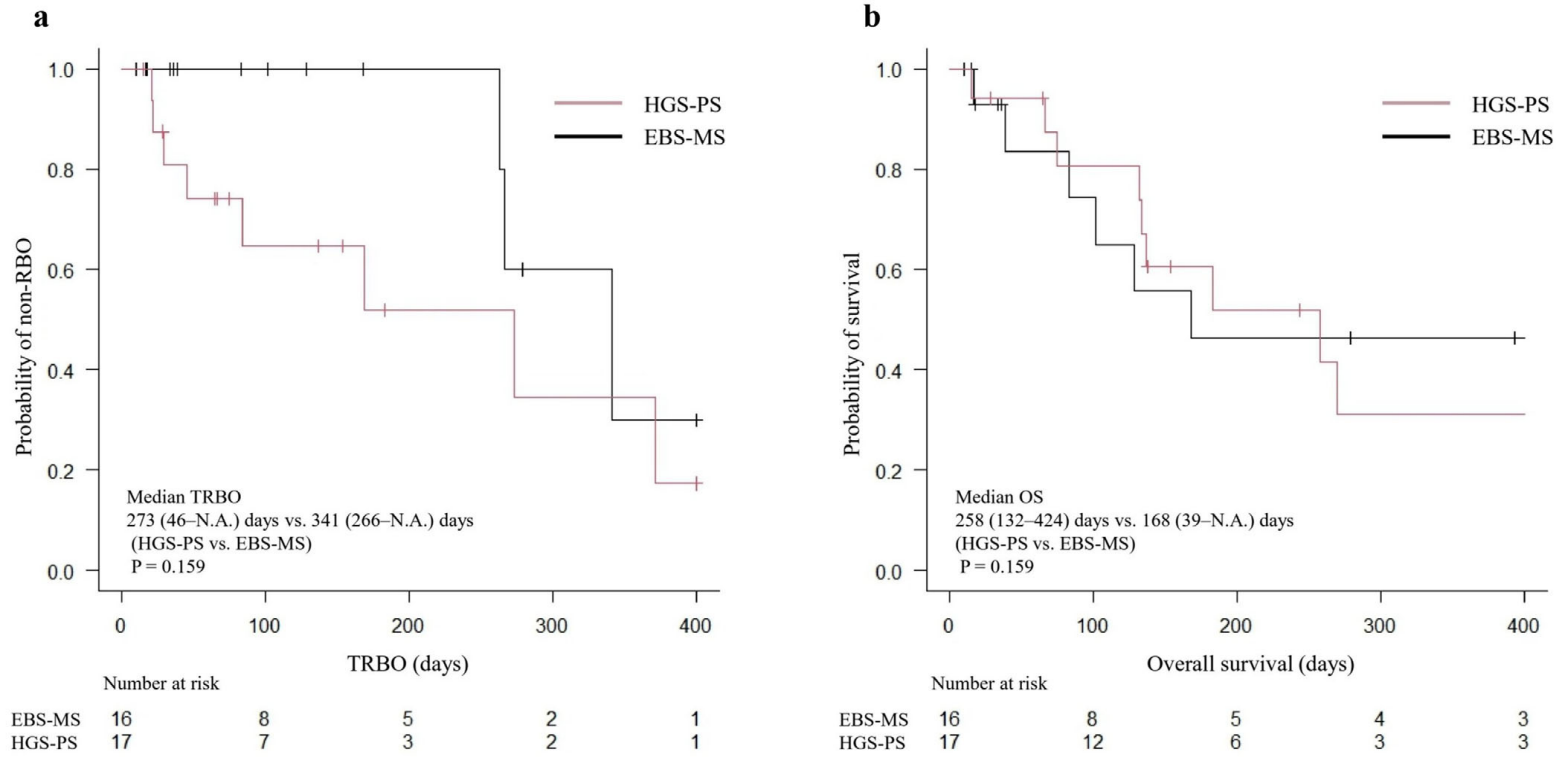
Kaplan‐Meier analysis of the probability of non‐RBOs and survival comparing the HGS‐PS and EBS‐MS groups of the propensity score‐matched cohort. (a) The median TRBO was 273 and 341 days in the HGS‐PS and EBS‐MS group, respectively, and there was no significant difference (*p* = 0.159). (b) The median OS was 258 and 168 days in the HGS‐PS and EBS‐MS group, respectively (*p* = 0.431). RBO, recurrent biliary obstruction; HGS‐PS, endoscopic ultrasound‐guided hepaticogastrostomy with a plastic stent; EBS‐MS, endoscopic retrograde cholangiopancreatography‐guided biliary stenting with a self‐expandable metallic stent; TRBO, time to recurrent biliary obstruction; OS, overall survival.

The median procedure time was significantly shorter in the HGS‐PS group compared to the ERCP group (24 vs. 45 min, *p* < 0.001). There were no significant differences in clinical success, RBO, and non‐RBO AEs. The median TRBO was 273 (46–not available [N.A.]) days for the HGS‐PS group and 341 (263–N.A.) days for the EBS‐MS group, with no significant difference (*p* = 0.159) (Figure 4a). There was no significant difference in the median OS of the two groups (258 [132–424] vs. 168 [39–N.A.] days, *p* = 0.431) (Figure 4b).

### 3.5 Subgroup Analyses

**FIGURE 5:**
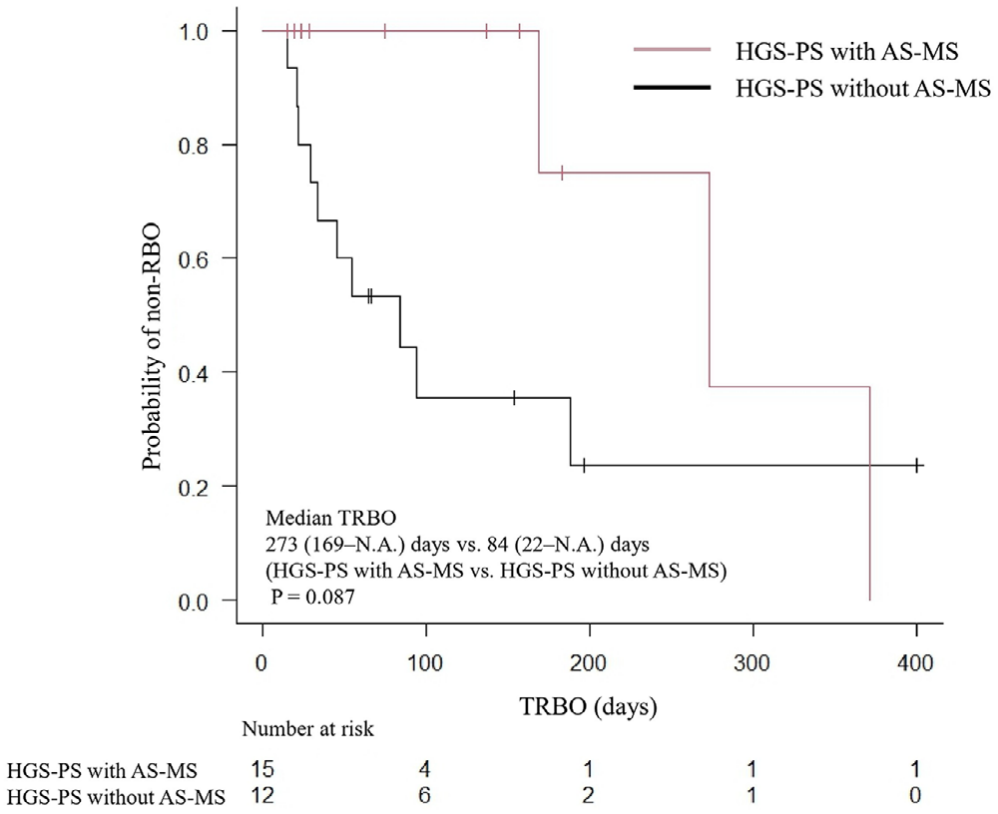
Kaplan‐Meier analysis of the probability of non‐RBO comparing the HGS‐PS with and without AS‐MS groups. The median TRBO was 273 and 84 days in the HGS‐PS with AS‐MS and without AS‐MS group, respectively (*p* = 0.087). RBO, recurrent biliary obstruction; HGS‐PS, endoscopic ultrasound‐guided hepaticogastrostomy with a plastic stent; AS‐MS, endoscopic ultrasound‐guided antegrade stenting with a self‐expandable metallic stent; TRBO, time to recurrent biliary obstruction.

A subgroup analysis was conducted comparing patients in the HGS‐PS group with (n = 12) and without AS‐MS (n = 15) (Table 5). The RBO rate was 25% with and 67% without AS‐MS (*p* = 0.102). The median TRBO was 273 (169–N.A.) days and 84 (22–N.A.) days, respectively (Figure 5). Although no significant difference in TRBO was observed (*p* = 0.087), AS‐MS showed a trend toward longer TRBO. No significant differences were observed in procedure time, non‐RBO AEs, and OS.

**TABLE 5:** Clinical outcomes of endoscopic ultrasound‐guided hepaticogastrostomy with plastic stents (HGS‐PS) with and without endoscopic ultrasound‐guided antegrade stenting with self‐expandable metallic stents (AS‐MS).

	HGS‐PS with AS‐MS	HGS‐PS without AS‐MS	*p*‐Value
	*n* = 12	*n* = 15	
Procedure time, minutes, median (range)	**27 (19–40)**	**19 (9–87)**	0.063
Clinical success, *n* (%)	12 (100)	15 (100)	N.A.
RBO, *n* (%)	3 (25)	10 (67)	0.102
TRBO, days, median (95%CI)	273 (169–N.A.)	84 (22–N.A.)	0.087
Non‐RBO AEs, *n* (%)	3 (25)	3 (20)	1.000
Acute pancreatitis	1 (8.3)	0	0.444
Peritonitis	2 (17)	3 (20)	1.000
OS, days, median (95%CI)	157 (20–258)	446 (134–N.A.)	0.105

AEs, adverse events; AS‐MS, endoscopic ultrasound‐guided antegrade stenting with self‐expandable metallic stents; CI, confidence intervals; HGS‐PS, endoscopic ultrasound‐guided hepaticogastrostomy with plastic stents; N.A., not available; OS, overall survival; RBO, recurrent biliary obstruction; TRBO, time to recurrent biliary obstruction.

**FIGURE 6:**
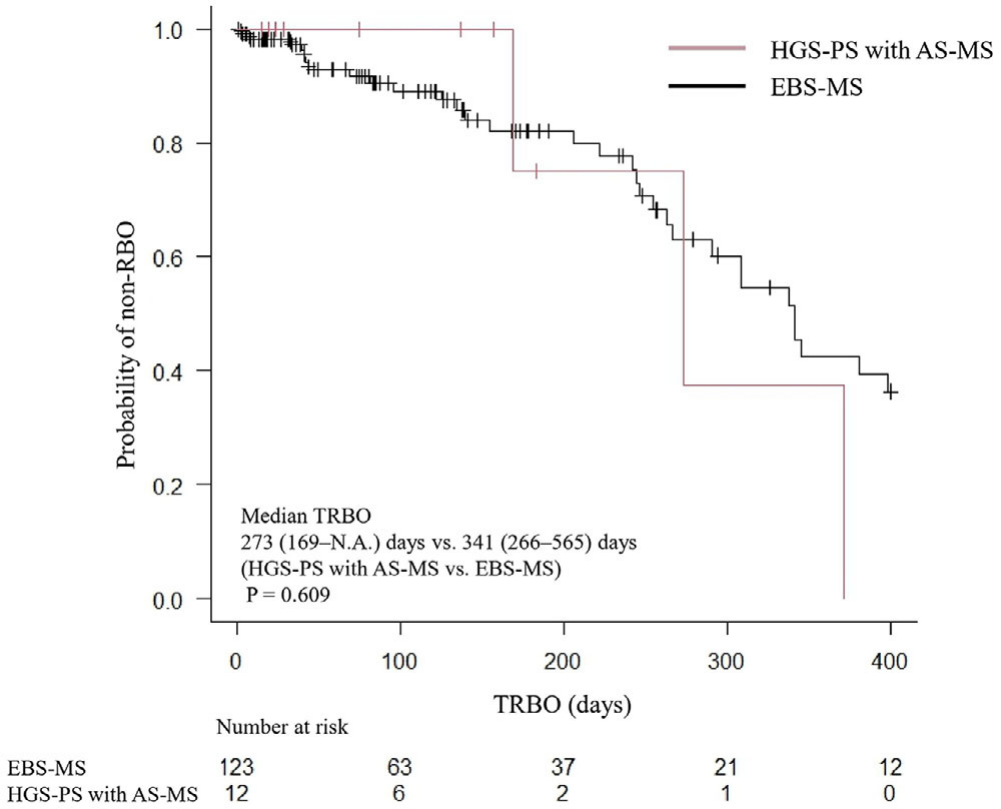
Kaplan‐Meier analysis of the probability of non‐RBOs comparing the HGS‐PS with AS‐MS and EBS‐MS groups. The median TRBO was 273 and 341 days in the HGS‐PS with AS‐MS and EBS‐MS groups, respectively (*p* = 0.609). RBO, recurrent biliary obstruction; HGS‐PS, endoscopic ultrasound‐guided hepaticogastrostomy with a plastic stent; AS‐MS, endoscopic ultrasound‐guided antegrade stenting with a self‐expandable metallic stent; EBS‐MS, endoscopic retrograde cholangiopancreatography‐guided biliary stenting with a self‐expandable metallic stent; TRBO, time to recurrent biliary obstruction.

Additionally, HGS‐PS with AS‐MS (*n* = 12) was compared with the EBS‐MS group (*n* = 128) (Table 6). The median procedure time was significantly shorter in the HGS‐PS group, even with the addition of AS‐MS (27 vs. 39 min, *p* = 0.029). The median TRBO did not differ significantly (273 [169–N.A.] vs. 341 [266–565] days, *p* = 0.609) (Figure 6), and other clinical outcomes were similar between groups.

**TABLE 6:** Clinical outcomes of endoscopic ultrasound‐guided hepaticogastrostomy with plastic stents (HGS‐PS) with endoscopic ultrasound‐guided antegrade stenting with self‐expandable metallic stents (AS‐MS) and endoscopic retrograde cholangiopancreatography‐guided biliary stenting with self‐expandable metallic stents (EBS‐MS).

	HGS‐PS with AS‐MS	EBS‐MS	*p*‐Value
	*n* = 12	*n* = 128	
Procedure time, minutes, median (range)	27 (19–40)	39 (9–101)	0.029
Clinical success, *n* (%)	12 (100)	123 (96)	0.588
RBO, *n* (%)	3 (25)	32 (26)	0.612
TRBO, days, median (95%CI)	273 (169–N.A.)	341 (266–565)	0.609
Non‐RBO AEs, *n* (%)	3 (25)	3 (20)	1.000
Acute pancreatitis	1 (8.3)	15 (12)	1.000
Cholecystitis	0	18 (14)	0.364
Nonocclusion cholangitis	0	2 (1.6)	1.000
Bleeding	0	5 (3.9)	1.000
Liver abscess	0	2 (1.6)	1.000
Perforation	0	1 (0.8)	1.000
Peritonitis	2 (17)	0	0.007
Death due to AEs, *n* (%)	0	1 (0.8)	1.000
OS, days, median (95% CI)	157 (20–258)	248 (171–327)	0.176

AEs, adverse events; AS‐MS, endoscopic ultrasound‐guided antegrade stenting with self‐expandable metallic stents; CI, confidence intervals; EBS‐MS, endoscopic retrograde cholangiopancreatography‐guided biliary stenting with self‐expandable metallic stents; HGS‐PS, endoscopic ultrasound‐guided hepaticogastrostomy with plastic stents; N.A., not available; OS, overall survival; RBO, recurrent biliary obstruction; TRBO, time to recurrent biliary obstruction.

## 4 Discussion

This is the first study comparing clinical outcomes of HGS‐PS and EBS‐MS as the initial biliary drainage for unresectable MDBO. The median procedure time was significantly shorter in the HGS‐PS group, and the incidence of non‐RBO AEs was comparable. Although TRBO tended to be shorter in the HGS‐PS group, the addition of AS‐MS made TRBO comparable to that of EBS‐MS.

In clinical practice, HGS‐PS has been performed less frequently than HGS‐MS, mainly because of its shorter TRBO and concerns about AEs. However, recent studies have demonstrated the clinical utility of HGS‐PS. Although TRBO is significantly longer when HGS‐MS is performed [7, 8], HGS‐PS is associated with a lower incidence of non‐RBO AEs compared to HGS‐MS [8]. In contrast to SEMSs, PSs can be placed with less concern for intrahepatic bile duct obstruction, resulting in a lower incidence of cholangitis or liver abscess. Furthermore, a major advantage of HGS‐PS is that reintervention is often technically less challenging due to the removability of PSs [8, 26]. With recent advances in chemotherapy and comprehensive genomic profiling, OS has improved [27, 28], and the need for reintervention is expected to increase. Therefore, selecting stents with consideration of future reintervention has become increasingly important, and the utility of PSs has been evaluated. In this context, we clarified the clinical outcomes of HGS‐PS in comparison with EBS‐MS.

There have been a few studies comparing EUS‐HGS and EBS‐MS as the initial drainage for unresectable MDBO, all of which have focused solely on HGS‐MS [19, 20, 21]. According to the studies, the incidence of non‐RBO AEs was 8.3%–48% in the HGS‐MS group and 9.1%–39% in the EBS‐MS group. TRBO was 171–366 days and 76–264 days, respectively. Based on these outcomes, HGS‐MS seems to offer clinical outcomes almost comparable to those of EBS‐MS. Although HGS‐MS may be particularly advantageous for patients with limited prognoses, for those undergoing chemotherapy with prolonged survival expected, treatment strategies incorporating HGS‐PS should be considered, given the possibility of reintervention.

The short TRBO of HGS‐PS is an issue to be addressed, and AS‐MS has recently been shown to improve outcomes. Ishiwatari et al. have reported that the median TRBO is significantly longer for the HGS‐MS with AS‐MS than for the HGS‐MS without AS‐MS (716 days vs. 194 days, *p* < 0.01) without an increased incidence of non‐RBO AEs [29]. Furthermore, the addition of AS‐MS is thought to have the potential to reduce the risk of peritonitis because bile flows through the major duodenal papilla after EUS‐AS. In our subgroup analysis comparing HGS‐PS with and without AS‐MS groups, AS‐MS appeared to extend TRBO even in the HGS‐PS group, although no statistically significant difference was observed due to the small sample size. HGS‐PS with AS‐MS can be regarded as a combination of EBS‐MS and an ESCR stent. Thus, TRBO was comparable as expected. However, AS‐MS may increase the risk of non‐RBO AEs, such as pancreatitis or cholecystitis. Since pancreatic atrophy and a dilated main pancreatic duct have been reported to be associated with a lower risk of post‐procedural pancreatitis [30], limiting AS‐MS to such patients may ensure safer HGS‐PS with longer TRBO.

This study has some limitations. First, this was a single‐center retrospective study with a limited sample size, particularly for the HGS‐PS group. EUS‐HGS techniques and devices evolved during the 7‐year study period, possibly influencing results. Second, in 16 of 27 HGS‐PS cases, the duodenoscope could not reach the papilla due to duodenal invasion, precluding equal opportunity for both approaches. Third, baseline heterogeneity in patient characteristics, such as primary disease or duodenal stenosis, may have influenced outcomes. Although PSM was conducted, the reduced cohort size may have resulted in insufficient statistical power. Fourth, the procedure time for the EBS‐MS group appears relatively long. In our institution, EBS‐MS is preferentially performed as the first‐line approach even in cases with suspected tumor invasion of the papilla, where biliary cannulation is expected to be difficult. These technically challenging cases may have contributed to the longer procedure time. Fifth, the indication for AS‐MS depended on the discretion of the endoscopist. Therefore, in some cases, even when the guidewire successfully passed through the stricture, AS‐MS may not have been performed due to concerns about post‐procedural pancreatitis. This limitation is inherent to the retrospective design.

In conclusion, as an initial biliary drainage for patients with unresectable MDBO, HGS‐PS alone was associated with shorter TRBO compared to EBS‐MS. However, the addition of AS‐MS to HGS‐PS resulted in TRBO similar to that of EBS‐MS, suggesting this combined approach may be a viable alternative.

## Author Contributions

Conceptualization: HS and YK. Data curation: HS, YK, SK, TO, HK, TS, KY, KM, FK, HO, KH, and SN. Formal analysis: HS. Investigation: HS, YK, SK, TO, HK, TS, KY, KM, FK, HO, KH, and SN. Methodology: HS and YK. Project administration: HS and YK. Supervision: YK and KI. Validation: YK and KI. Visualization: HS and YK. Writing–original draft: HS and YK. Writing–review & editing: all authors.

## Conflicts of Interest

The authors declare no conflicts of interest.

## Funding

The authors have nothing to report.

## Ethics Statement

**Approval of the research protocol by an Institutional Reviewer Board**: This study was approved by the institutional review board of Sendai City Medical Center (approval number 2024‐0064).

## Consent

Written informed consent for the procedures was obtained from all patients, and participation in this study was approved through an opt‐out process on the hospital website.
